# Medication belief profiles and associated factors in patients receiving maintenance hemodialysis: a latent class analysis

**DOI:** 10.3389/fpubh.2026.1855187

**Published:** 2026-07-22

**Authors:** Fengxue Yang, Yan Zhong, Li Zeng, Zhongqing Yuan, Yin Zhou, Yi Tian

**Affiliations:** 1 Sichuan Nursing Vocational College, Chengdu, Sichuan, China; 2 State Key Laboratory of Oral Diseases, National Center for Stomatology, National Clinical Research Center for Oral Diseases and Other Research Platforms, The Eastern District Outpatient Department, West China Hospital of Stomatology Sichuan University, Chengdu, Sichuan, China; 3 Innovation Center of Nursing Research and Nursing Key Laboratory of Sichuan Province, West China Hospital, West China School of Nursing, Sichuan University, Chengdu, Sichuan, China; 4 Chengdu Second People’s Hospital, Sichuan University, Chengdu, Sichuan, China

**Keywords:** health education, latent class analysis, maintenance hemodialysis, medication beliefs, perceived medication knowledge

## Abstract

**Background:**

Medication beliefs play an important role in how patients understand and manage long-term treatment. Among patients receiving maintenance hemodialysis, identifying clinically meaningful patterns of medication beliefs may help inform more targeted and patient-centered medication management strategies.

**Methods:**

A cross-sectional study was conducted among maintenance hemodialysis patients using the Beliefs about Medicines Questionnaire–Specific (BMQ-Specific) and the Patient-Perceived Medication Knowledge in Medication Use Scale (Okere–Renier Survey). Latent class analysis (LCA) was applied to identify distinct medication belief profiles. Demographic characteristics, medication-related variables, and perceived medication knowledge were compared across latent classes, and multivariable logistic regression was used to identify factors associated with belief profile membership.

**Results:**

Three distinct medication belief profiles were identified: a Poor Overall Medication Beliefs Group (34.5%), a Higher Concern Beliefs Group (33.3%), and a Better Overall Medication Beliefs Group (32.2%). Classification among these categories of medication beliefs was linked to gender, medication usage frequency, family economic level, and knowledge on medication.

**Conclusion:**

Patients on maintenance hemodialysis show varied and medically relevant medication beliefs. The correlation between these beliefs and demographic factors, as well as factors concerning their medication regimen, shows that there is an additional requirement for tailored medication education in hemodialysis patients.

## 1 Introduction

Patients on maintenance hemodialysis need to have complicated medication regimens over the long run to help address the issues and comorbid conditions they might have. Medication beliefs are an important factor influencing how patients understand, evaluate, and manage their prescribed treatments. Patients who hold inaccurate or negative beliefs about medications may be less likely to perceive their treatment as necessary and may experience greater concerns about potential adverse effects, which can negatively affect treatment engagement and medication management (1, 2). Therefore, understanding medication beliefs is important for supporting shared decision-making, patient engagement, and individualized medication management (3).

Studies have shown that misconceptions about treatment need and adverse reactions can greatly influence the level of compliance among patients, especially when taking prescribed medictaions like insulin in diabetes (4). With respect to maintenance hemodialysis, non-compliance with medications continues to be an issue despite the heavy medication load and long duration of treatment (5). Studies have proven that patients’ beliefs on medications correlate directly with their perception, usage, and management of prescribed medications, which apply to patients on hemodialysis too (6–8). Nonetheless, very little information is available with regard to distinct belief patterns in patients on hemodialysis and their correlations with patients’ demographics and medications.

Latent class analysis (LCA) offers a person-centered analytic approach that enables the identification of unobserved subgroups based on shared response patterns (9, 10). Rather than assuming that all patients hold similar beliefs about treatment, LCA can be used to identify clinically meaningful subgroups that may differ in their perceptions, concerns, and support needs. Previous studies have applied LCA to explore heterogeneity in health-related behaviors and perceptions in patients with chronic conditions (11).

Applying this approach, the present study aimed to identify distinct medication belief profiles among patients receiving maintenance hemodialysis using latent class analysis and to examine their associations with demographic, clinical, and medication-related factors. By characterizing belief-based subgroups, this study may provide evidence to support more targeted and patient-centered strategies for medication education and management in routine dialysis care.

## 2 Methods

### 2.1 Study sample

This cross-sectional study was conducted in March 2025. Participants were eligible if they were aged ≥18 years and had been receiving maintenance hemodialysis for at least 3 months. Patients were recruited during routine dialysis sessions. Individuals with cognitive impairment or mental disorders that could interfere with questionnaire completion were excluded.

The study was approved by the Medical Ethics Committee of Sichuan Nursing Vocational College (Approval no. 2025010) and was conducted in accordance with the Declaration of Helsinki (2013 revision). Written informed consent was obtained from all participants prior to data collection.

### 2.2 Sample size

This study used multivariable logistic regression to examine factors associated with medication belief profiles among maintenance hemodialysis patients. Nineteen independent variables were initially proposed based on a literature review. According to the principle that the sample size should be 5 to 10 times the number of independent variables, and allowing for a potential 20% rate of invalid responses, the required sample size was estimated to range from 114 to 228 participants.

### 2.3 Survey instrument

#### 2.3.1 Demographic and medication-related characteristics

A self-designed questionnaire was used to collect demographic information, including age, gender, education level, marital status, employment status after illness, place of residence, and source of income. Medication-related variables included the number of prescribed medications, medication frequency, and duration of medication use.

#### 2.3.2 Beliefs about Medicines Questionnaire (BMQ-specific)

The Beliefs about Medicines Questionnaire–Specific (BMQ-Specific), developed by Horne et al. (12), was used to assess patients’ beliefs about their prescribed medications. The BMQ-Specific comprises two five-item subscales evaluating patients’ beliefs about the necessity of prescribed medications and their concerns about potential adverse consequences. These two dimensions reflect how patients evaluate the perceived benefits and possible risks of long-term medication use, which is particularly relevant for patients receiving maintenance hemodialysis. Responses are rated on a 5-point Likert scale ranging from 1 (“strongly disagree”) to 5 (“strongly agree”).

The Chinese version of the BMQ-Specific has demonstrated good reliability and validity. In this study, the Cronbach’s alpha coefficients were 0.833 for the necessity beliefs subscale and 0.845 for the concern beliefs subscale. For latent class analysis, each item was dichotomized in accordance with previous research (13), with responses of “strongly disagree,” “disagree,” and “uncertain” coded as 0, and responses of “agree” and “strongly agree” coded as 1. The total medication belief score was calculated as the difference between the necessity and concern subscale scores.

#### 2.3.3 Patient-Perceived Medication Knowledge in Medication Use Scale (Okere–Renier survey score)

Patients’ perceived medication knowledge was measured using the Patient-Perceived Medication Knowledge in Medication Use Scale, also known as the Okere–Renier Survey, developed by Okere et al. (14). This self-report instrument assesses patients’ perceived medication knowledge, with items addressing general medication knowledge, such as how and when to take medications, as well as understanding of medication interactions and possible side effects. The knowledge scale comprises five items, each rated on a 5-point Likert scale with response options ranging from “strongly disagree” to “strongly agree.” Because medication knowledge may shape how patients understand and evaluate the necessity, benefits, and risks of long-term medication use, perceived medication knowledge was included as a medication-related factor potentially associated with medication belief profiles. The scale has demonstrated good psychometric properties, with a Cronbach’s alpha coefficient of 0.833.

### 2.4 Analysis

Data were analyzed using SPSS version 25.0 (IBM Corp., Armonk, NY, United States) and Mplus version 8.0. Descriptive statistics were used to summarize participant characteristics. Normality of continuous variables was assessed using the Kolmogorov–Smirnov test. Continuous variables with non-normal distributions are presented as median and interquartile range, and between-group comparisons were conducted using the Kruskal–Wallis H test. Categorical variables are presented as number (percentage) and were compared using the Pearson chi-square test or Fisher–Freeman–Halton test, as appropriate (15).

Spearman correlation analysis was performed to examine associations between the total medication belief score and the necessity and concern subscale scores. Latent class analysis (LCA) was conducted using Mplus to identify distinct medication belief profiles. Models with increasing numbers of classes were estimated sequentially. Model fit was evaluated using the Akaike Information Criterion (AIC), Bayesian Information Criterion (BIC), sample-size adjusted BIC (SSA-BIC), entropy, the Lo–Mendell–Rubin likelihood ratio test (LMR-LRT), and the bootstrap likelihood ratio test (BLRT). Lower information criterion values, entropy ≥0.80, and statistically significant LMR-LRT and BLRT results were considered indicative of better model fit (16–20).

Multivariable logistic regression analysis was subsequently performed to identify factors associated with latent medication belief class membership. A two-sided *p* value <0.05 was considered statistically significant.

## 3 Results

### 3.1 Sample characteristics

The study included 177 patients receiving maintenance hemodialysis. The mean age was 47.3 ± 11.98 years, and 110 participants (62.1%) were women. Sixteen participants (9.0%) had a primary school education or below, and 110 (62.1%) were single. The mean dialysis vintage was 4.91 ± 3.76 years. Fourteen participants (7.9%) had a history of kidney transplantation. Regarding medication-related characteristics, 103 participants (58.2%) were prescribed 4–6 medications, and 70 (39.5%) reported taking medications twice daily. Hypertension (79.7%), anaemia (73.4%), and hyperuricemia (45.2%) were the most common comorbidities in the study population (Table 1).

**Table 1:** Sample characteristics of the study participants.

Variables		Frequencies (%)	Mean (SD)/ median (Range)
Gender	Female	110 (62.1%)	
	Male	67 (37.9%)	
Age			47.3 (11.98)
Marital status	Single	110 (62.1%)	
	Married	67 (37.9%)	
Education	Primary school or lower	16 (9.0%)	
	Junior high school	53 (29.9%)	
	High school or senior high school	49 (27.7%)	
	College or higher	59 (33.3%)	
Home location	Large cities	97 (54.8%)	
	Countryside	45 (25.4%)	
	Small cities or town	35 (19.8%)	
Occupational status	Unemployed	86 (48.6%)	
	Employed	55 (31.1%)	
	Retired	36 (20.3%)	
Family economic status	None	25 (14.1%)	
	Light	28 (15.8%)	
	Medium	61 (34.5%)	
	Heavy	63 (35.6%)	
Frequency of medication use	Once daily	41 (23.2%)	
	Twice daily	70 (39.5%)	
	Three or more times daily	66 (37.3%)	
Number of medications	1–3	52 (29.4%)	
	4–6	103 (58.2%)	
	7–9	16 (9.0%)	
	≥10	6 (3.4%)	
Dialysis vintage (yrs)			4.91 (3.76)
History of kidney transplantation	Yes	14 (7.9%)	
	No	162 (91.5%)	
Comorbidities	Anaemia	130 (73.4%)	
	Cardiovascular disease	60 (33.9%)	
	Hypertension	141 (79.7%)	
	Diabetes	21 (11.9%)	
	Gastrointestinal disease	26 (14.7%)	
	Hyperkalemia	48 (27.1%)	
	Hyperuricemia	80 (45.2%)	
	Anxiety, depression, or insomnia	39 (22.0%)	
BMQ-specific score			5.47 (3.59)
Necessity beliefs score			20.89 (3.75)
Concerns beliefs score			15.42 (3.37)
Okere–renier survey score			19.39 (5.03)

### 3.2 BMQ-specific and Okere–Renier survey scores

Among the 177 patients receiving maintenance hemodialysis, the mean BMQ-Specific score was 5.47 ± 3.59, with mean necessity beliefs and concerns beliefs scores of 20.89 ± 3.75 and 15.42 ± 3.37, respectively. The mean Okere–Renier Survey score was 19.39 ± 5.03 (Table 1).

### 3.3 Latent class analysis of medication beliefs

Model fit statistics for the 2-class to 5-class models are presented in Table 2. The 4-class and 5-class models showed non-significant LMR and adjusted LMR test results, whereas the 3-class model demonstrated acceptable fit, with lower BIC and sample-size adjusted BIC values than the 2-class model and an entropy value of 0.802. Considering model fit, class separation, interpretability, and class sizes, the 3-class model was selected as the optimal solution.

**Table 2:** Model fit indices for latent class analysis of medication beliefs.

Model	k	Log-likelihood	AIC	BIC	ssaBIC	LMR *p* value	ALMR *p* value	BLRT p value	Entropy	Class proportion
2-class	21	−931.658	1905.316	1972.015	1905.512	0.0071	0.0077	<0.0001	0.798	0.48/0.52
**3-class**	**32**	**−891.383**	**1846.765**	**1948.402**	**1847.064**	**0.0202**	**0.0214**	**<0.0001**	**0.802**	**0.34/0.33/0.32**
4-class	43	−867.641	1821.283	1957.857	1821.684	0.3276	0.3324	<0.0001	0.801	0.32/0.13/0.25/0.29
5-class	54	−853.67	1815.34	1986.852	1815.844	0.2241	0.2275	0.04	0.83	0.16/0.21/0.23/0.24/0.14

Bold values indicate the fit indices of the selected optimal model.

**Figure 1:**
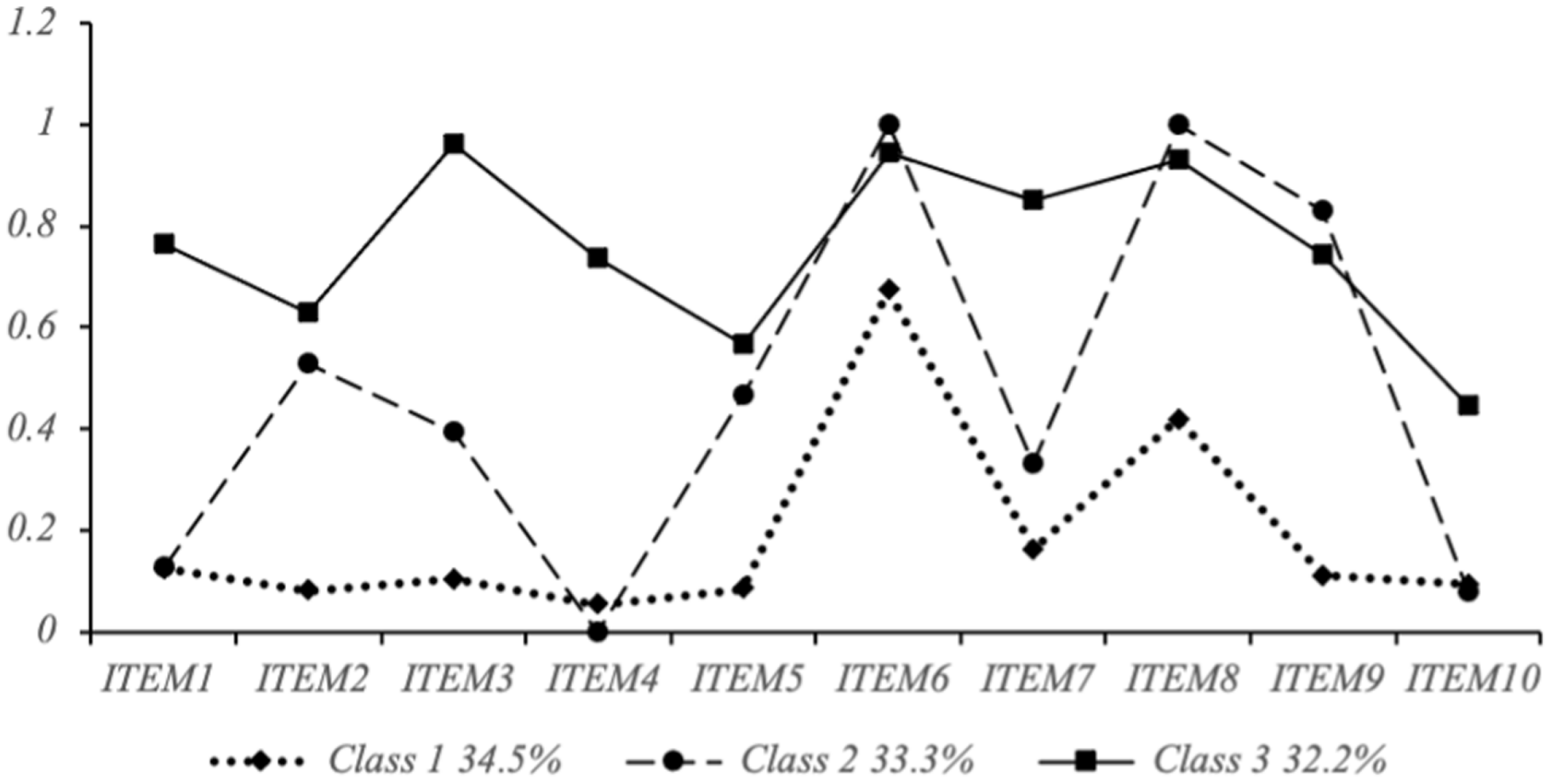
Conditional response probabilities for the three latent classes of medication beliefs among maintenance hemodialysis patients. Item 1: My health, at present, depends on my medicines Item 2: Having to take these medicines worries me Item 3: My life would be impossible without my medicines Item 4: Without my medicines I would be very ill Item 5: I sometimes worry about long-term effects of my medicines Item 6: My medicines are a mystery to me Item 7: My health in the future will depend on my medicines Item 8: My medicines disrupt my life Item 9: I sometimes worry about becoming too dependent on my medicines Item 10: My medicines protect me from becoming worse.

The conditional probability distributions for the three latent classes are shown in Figure 1. Class 1 comprised 61 participants (34.5%), Class 2 comprised 59 participants (33.3%), and Class 3 comprised 57 participants (32.2%).

Based on the conditional response probabilities, Class 3 showed consistently high probabilities across most BMQ-Specific items and was therefore labeled the “Better Overall Medication Beliefs Group.” Class 2 showed relatively elevated probabilities for several concern-related items, particularly items 6, 8, and 9, and was labeled the “Higher Concern Beliefs Group.” In contrast, Class 1 showed generally low conditional probabilities across items and was therefore labeled the “Poor Overall Medication Beliefs Group.”

### 3.4 Comparison of characteristics across medication belief groups

Comparisons across the three medication belief groups showed statistically significant differences in gender, family economic status, frequency of medication use, and number of medications (all *p* < 0.05). No significant differences were observed for age, marital status, education, home location, occupational status, dialysis vintage, history of kidney transplantation, comorbidity, or Okere–Renier Survey score (Table 3).

**Table 3:** Comparison of the characteristics of maintenance hemodialysis patients with different medication belief groups.

Variables		Class 1:	Class 2:	Class 3:	Chi-square or H test	
		Poor overall medication beliefs group	Higher concern beliefs group	Better overall medication beliefs group		*p*-value
		(*N* = 61, 34.5%)	(*N* = 59, 33.3%)	(*N* = 57, 32.2%)		
Demographic variables						
Gender	Female	35 (57.4%)	35 (59.3%)	40 (70.2%)	5.957^a^	0.015
	Male	26 (42.6%)	24 (40.7%)	17 (29.8%)		
Age	<40 years old	13 (21.3%)	18 (30.5%)	23 (40.4%)	9.909^a^	0.129
	40 ~ 49 years old	14 (23.0%)	20 (33.9%)	12 (21.1%)		
	50 ~ 59 years old	24 (39.3%)	13 (22.0%)	13 (22.8%)		
	≥ 60 years old	10 (16.4%)	8 (13.6%)	9 (15.8%)		
Marital status	Single	35 (57.4%)	35 (59.3%)	40 (70.2%)	2.352^a^	0.309
	Married	26 (42.6%)	24 (40.7%)	17 (29.8%)		
Education	Primary school or lower	10 (16.4%)	2 (3.4%)	4 (7.0%)	9.847^a^	0.132
	Junior high school	19 (31.1%)	15 (25.4%)	19 (33.3%)		
	High school or senior high school	12 (19.7%)	19 (32.2%)	18 (31.6%)		
	College or higher	20 (32.8%)	23 (39.0%)	16 (28.1%)		
Home location	Large cities	30 (49.2%)	39 (66.1%)	28 (49.1%)	7.214^a^	0.126
	Countryside	20 (32.8%)	8 (13.6%)	17 (29.8%)		
	Small cities or town	11 (18.0%)	12 (20.3%)	12 (21.1%)		
Occupational status	Unemployed	34 (55.7%)	27 (45.8%)	25 (43.9%)	2.301^a^	0.688
	Employed	15 (24.6%)	20 (33.9%)	20 (35.1%)		
	Retired	12 (19.7%)	12 (20.3%)	12 (21.1%)		
Family economic status	None	5 (8.2%)	13 (22.0%)	7 (12.3%)	13.21^a^	0.039
	Light	9 (14.8%)	9 (15.3%)	10 (17.5%)		
	Medium	16 (26.2%)	20 (33.9%)	25 (43.9%)		
	Heavy	31 (50.8%)	17 (28.8%)	15 (26.3%)		
Frequency of medication use	Once daily	6 (9.8%)	14 (23.7%)	21 (36.8%)	12.561^a^	0.013
	Twice daily	27 (44.3%)	25 (42.4%)	18 (31.6%)		
	Three or more times daily	28 (45.9%)	20 (33.9%)	18 (31.6%)		
Number of medications	1–3	10 (16.4%)	15 (25.4%)	27 (47.4%)	19.700^b^	0.001
	4–6	39 (63.9%)	41 (69.5%)	23 (40.4%)		
	7–9	9 (14.8%)	2 (3.4%)	5 (8.8%)		
	≥10	3 (4.9%)	1 (1.7%)	2 (3.5%)		
Dialysis vintage (yrs)	5.51 (4.100)	4.41 (3.469)	4.80 (3.655)	2.278^c^	0.32	
History of kidney transplantation	Yes	5 (8.2%)	3 (5.1%)	6 (10.5%)	1.190^a^	0.054
	No	55 (90.2%)	56 (94.9%)	51 (89.5%)		
Number of comorbidities	<5	34 (55.7%)	43 (72.9%)	38 (66.7%)	3.979^a^	0.146
	≥5	27 (44.3%)	16 (27.1%)	19 (33.3%)		
Okere–renier survey score		20.21 (4.309)	20.31 (4.65)	17.56 (5.69)	6.441^c^	0.090

a, Pearson chi-square; b, Fisher’s exact test; c, Kruskal-wallis H test.

### 3.5 Multivariate logistic regression analysis of factors affecting the category of maintenance hemodialysis patients

Using the Better Overall Medication Beliefs Group (Class 3) as the reference category, multivariable logistic regression analysis showed that gender, frequency of medication use, family economic status, and Okere–Renier Survey score were associated with medication belief group membership (Table 4).

**Table 4:** Multivariate logistic regression analysis of three potential medication beliefs in maintenance hemodialysis patients.

	Class 1			Class 2	
Variables	Poor overall medication beliefs group			Higher concern beliefs group	
		OR	CI (95%)	OR	CI (95%)
Gender	Female	0.154*	0.132–0.910	0.152*	0.032–0.736
	Male	1		1	
Frequency of drug use	Once daily	0.269*	0.084–0.862	0.651	0.223–1.902
	Twice daily	1.331	0.491–3.606	1.285	0.474–3.487
	Three or more times daily	1		1	
Family economic status	None	0.303	0.061–1.510	1.235	0.308–4.955
	Light	0.319	0.083–1.224	0.561	0.149–2.119
	Medium	0.192*	0.064–0.575	0.569	0.196–1.649
	Heavy	1		1	
Okere–renier survey score		1.098*	1.008–1.196	1.094*	1.005–1.190

* *p* < 0.05.

Compared with male patients, female patients were less likely to be classified into the Poor Overall Medication Beliefs Group (Class 1) (OR = 0.154, 95% CI: 0.132–0.910) and the Higher Concern Beliefs Group (Class 2) (OR = 0.152, 95% CI: 0.032–0.736).

Compared with patients taking medications three or more times daily, those taking medications once daily were less likely to be classified into the Poor Overall Medication Beliefs Group (Class 1) (OR = 0.269, 95% CI: 0.084–0.862). No significant association was observed between frequency of medication use and membership in the Higher Concern Beliefs Group (Class 2).

Compared with patients reporting heavy family economic burden, those reporting a moderate economic burden were less likely to be classified into the Poor Overall Medication Beliefs Group (Class 1) (OR = 0.192, 95% CI: 0.064–0.575). Family economic status was not significantly associated with membership in the Higher Concern Beliefs Group (Class 2).

Higher Okere–Renier Survey scores were associated with an increased likelihood of belonging to both the Poor Overall Medication Beliefs Group (Class 1) (OR = 1.098, 95% CI: 1.008–1.196) and the Higher Concern Beliefs Group (Class 2) (OR = 1.094, 95% CI: 1.005–1.190).

## 4 Discussion

This study identified three distinct and clinically meaningful medication belief profiles among patients receiving maintenance hemodialysis: a Poor Overall Medication Beliefs Group (C1), a Higher Concern Beliefs Group (C2), and a Better Overall Medication Beliefs Group (C3). These belief profiles were associated with demographic characteristics, medication-related factors, and perceived medication knowledge. By applying a person-centered analytic approach, this study extends existing evidence by demonstrating that medication beliefs among hemodialysis patients are heterogeneous and may require differentiated management strategies rather than uniform educational interventions.

### 4.1 Heterogeneity in medication belief among maintenance dialysis patients

Latent class analysis in the current study revealed substantial heterogeneity in medication beliefs among maintenance hemodialysis patients. Approximately one-third of patients were classified into each belief profile, with 34.5% exhibiting poor overall medication beliefs, 33.3% demonstrating higher concern beliefs, and 32.2% showing better overall medication beliefs toward their prescribed medications. This finding supports the earlier studies conducted on chronic kidney disease patients, wherein it was found that even though majority of patients considered the medicines prescribed to them as necessary, there was considerable portion of patients who had high concerns with regards to medication therapy (21).

Patients in the Better Overall Medication Beliefs Group demonstrated stronger perceived necessity and lower concern regarding medication use, suggesting greater acceptance of long-term pharmacological treatment. In contrast, patients in the Higher Concern Beliefs Group appeared to experience substantial apprehension regarding medication use. This pattern aligns with previous findings by Drangsholt et al., who reported that although most dialysis patients do not perceive medicines as harmful, a considerable proportion believe that medicines are overprescribed and remain uncertain about their long-term effects (22). Worrying about medicines may, in some way, stem from the understanding that patients have regarding their condition and medicines (23). Patients who understand more clearly their illness and the need for taking medicines over a period of time will have more perception about the necessity of the medication prescribed to them. On the other hand, a lack of understanding regarding the illness or disease threat may lead to worrying with regards to taking medicines.

The Poor Overall Medication Beliefs Group accounted for more than one-third of the sample, indicating that a substantial proportion of maintenance hemodialysis patients may lack confidence in the necessity and effectiveness of their medications. Similar findings have been reported in other chronic disease populations, where insufficient medication knowledge and low perceived treatment benefit were associated with unfavorable medication beliefs (24). From the perspective of empowerment theory, effective self-management requires patients to understand their responsibilities, strengthen their internal motivation, and enhance their self-efficacy (25). For patients with poor medication beliefs, healthcare professionals should focus on improving medication knowledge, correcting misconceptions, and supporting more positive engagement with long-term treatment.

### 4.2 Factors associated with medication belief profiles

Demographic and medication factors were found to correlate with medication belief group profiles.

Compared with male patients, female patients were less likely to be classified into the Poor Overall Medication Beliefs Group or the Higher Concern Beliefs Group, using the Better Overall Medication Beliefs Group as the reference category. This finding suggests that, in this sample, male patients may be more likely to exhibit less favorable medication belief patterns. This result is partly consistent with previous evidence showing that women generally have higher utilization of preventive healthcare services than men (26). The possible reason is that women are more sensitive to health risks and are therefore more willing to access healthcare resources, making them better equipped for handling diseases.

The financial burden also emerged as an important factor influencing medication beliefs. Patients reporting heavier financial burden were more likely to exhibit poorer medication beliefs. Cost-related concerns may lead patients to question the necessity or sustainability of long-term treatment, a phenomenon widely documented in chronic disease management (25). Considering the long-term and expensive character of hemodialysis, the financial burden may become a real impediment to forming favorable medication beliefs.

Medication frequency was another influential factor. Compared with patients taking medications three or more times daily, those taking medications once daily were less likely to belong to the Poor Overall Medication Beliefs Group. This finding may reflect differences in treatment burden, regimen complexity, or patients’ perceptions of treatment intensity. Consistent with self-regulation theory, patients’ beliefs may shape how they interpret treatment information and subsequently influence their attitudes toward medication use (27). This statement finds support in the findings of Horne et al. who argued that patients’ beliefs about medicines prescribed to them play a critical role in medication adherence (12). Therefore, simplification of the medication regimen when possible and assisting patients to comprehend the gravity of the disease as well as the necessity of long-term pharmacotherapy should be taken into account.

### 4.3 Perceived medication knowledge and belief patterns

Perceived medication knowledge, as measured by the Okere–Renier Survey, was significantly associated with both the Poor Overall Medication Beliefs Group and the Higher Concern Beliefs Group in multivariable analysis. Although the Okere–Renier Survey score did not differ significantly across groups in univariate analysis, the adjusted regression model suggested that perceived medication knowledge was independently associated with medication belief profile membership.

It is worth mentioning that higher perceived medication knowledge was associated with an increased likelihood of belonging to the Poor Overall Medication Beliefs Group or the Higher Concern Beliefs Group, using the Better Overall Medication Beliefs Group as the reference category. This finding suggests that greater perceived medication knowledge does not necessarily translate into more favorable medication beliefs. Patients who perceive themselves as more knowledgeable about medication use, medication interactions, and possible side effects may also be more aware of the complexity, potential risks, and long-term burden of pharmacotherapy, which could contribute to greater concern or ambivalence toward medication use. This may be related to greater medication-related concerns, negative perceptions of medication use, and perceived illness burden, which may contribute to less favorable medication beliefs. Therefore, helping patients develop an appropriate understanding of their disease and treatment may support active engagement in care, promote healthy behaviors, improve emotional responses to illness, and facilitate more effective coping strategies and medication management (28).

These findings highlight the importance of addressing not only patients’ medication knowledge but also the emotional and cognitive responses associated with long-term medication use (29, 30). Failure to recognize patients’ concerns and misunderstandings may negatively affect their treatment experience and long-term engagement with care.

### 4.4 Clinical relevance

The identification of distinct medication belief profiles provides a practical framework for improving medication management in maintenance hemodialysis settings. Belief-based profiling may help healthcare professionals recognize different patient needs and deliver more targeted support. Patients with poor overall medication beliefs may benefit from structured medication education and interventions that strengthen self-efficacy and treatment understanding, whereas those with higher concern beliefs may require individualized counseling to address worries about long-term medication use, adverse effects, and treatment burden. For patients with better overall medication beliefs, ongoing monitoring and periodic reassessment may help maintain positive engagement with treatment. Incorporating brief assessments of medication beliefs into routine dialysis care may support earlier identification of patients who need additional education or counseling and improve the efficiency of patient-centered medication management.

### 4.5 Study limitations and future directions

The study has many limitations. First, the cross-sectional design precludes causal inference, and the stability of medication belief profiles over time remains unknown. Because this study did not include longitudinal follow-up, we could not examine whether medication belief profiles were associated with subsequent clinical outcomes. Second, medication beliefs and perceived medication knowledge were assessed using self-reported measures, which may be subject to recall and social desirability bias. Third, because participants were recruited from a limited geographical area, the sample size was relatively small, and the participants were relatively young compared with the broader maintenance hemodialysis population, the findings may not be generalizable to maintenance hemodialysis patients in other regions, healthcare settings, or older age groups. Fourth, frailty, laboratory parameters, dialysis adequacy, and nutritional indicators were not systematically assessed, although these factors may influence medication beliefs, perceived medication knowledge, quality of life, and medication management among maintenance hemodialysis patients. Future research should include larger, multicenter, and more diverse samples, incorporate frailty assessment, include objective clinical indicators, and use longitudinal designs to validate these findings and evaluate their clinical prognostic significance.

## 5 Relevance for clinical practice

Distinct medication belief patterns among maintenance hemodialysis patients may have important implications for clinical practice. Latent class analysis provides a useful approach to identifying subgroups with poor overall or higher concern beliefs, who may benefit from more targeted educational and counseling interventions. Strengthening the assessment of medication beliefs in routine clinical practice may help healthcare professionals tailor nursing support, improve treatment understanding, and promote more patient-centered medication management in this population.

## 6 Conclusion

This study suggests that medication beliefs among maintenance hemodialysis patients are heterogeneous and can be classified into three distinct profiles using latent class analysis. These profiles were differentially associated with demographic characteristics, medication-related factors, and perceived medication knowledge. Recognizing belief-based differences may help inform the development of more targeted and patient-centered strategies for medication education and management in hemodialysis care.

## Data Availability

The datasets presented in this article are not readily available because the dataset is restricted due to privacy and ethical considerations. Because the data were collected from human participants, they are not available for sharing. Requests to access the datasets should be directed to fengxue.yang12@gmail.com.
